# Temporal progression of bone loss, trabecular microarchitectural deterioration, and mechanical impairment in a rat model of collagen-induced arthritis

**DOI:** 10.3389/fmed.2026.1885782

**Published:** 2026-08-17

**Authors:** Xuan Cheng, Yun Ling, Jun Xie, Lianbo Xiao, Xiong Shi, Chenxin Gao

**Affiliations:** 1 Department of Joint Orthopedics, Guanghua Hospital Affiliated to Shanghai, University of Traditional Chinese Medicine, Shanghai, China; 2 Graduate School, Shanghai University of Traditional Chinese Medicine, Shanghai, China

**Keywords:** bone loss, collagen-induced arthritis, inflammation, osteoporosis, rheumatoid arthritis

## Abstract

**Aim:**

This study aimed to characterize the temporal progression of bone loss, trabecular microarchitectural deterioration, and mechanical impairment in a rat model of collagen-induced arthritis (CIA).

**Methods:**

CIA was induced in Wistar rats via subcutaneous injection of bovine type II collagen. Following CIA induction, rats with a clinical arthritis score of ≥2 were considered to have successfully developed arthritis and were allocated to six experimental groups (*n* = 8 per group) for tissue collection at 2, 3, 4, 6, 8, and 10 weeks after enrollment. In parallel, three non-immunized normal control rats were euthanized at each corresponding observation time point. Bone quality was assessed using micro-computed tomography (micro-CT) of the proximal tibia and biomechanical testing of the femur and L5 vertebrae.

**Results:**

Micro-CT analysis revealed a significant decrease in bone mineral density (BMD) and trabecular thickness (Tb.Th) as early as 2 weeks post-induction (*p* < 0.05). After multiplicity correction, trabecular number (Tb.N) and trabecular separation (Tb.Sp) did not differ significantly between the model and corresponding normal groups at any individual time point. Biomechanical testing demonstrated significant reductions in both femoral bending strength and vertebral compressive strength from the earliest examined model time point at 2 weeks, and these impairments persisted through the final 10-week observation point.

**Conclusion:**

CIA induced early deterioration of bone mass and trabecular microarchitecture, characterized by significant reductions in BMD and Tb.Th. No statistically significant between-group differences in Tb.N or Tb.Sp were identified at individual time points after multiplicity correction. Mechanical impairment of both femoral and vertebral bone was also observed during disease progression. These findings indicate that microarchitectural deterioration and loss of bone strength may follow partly distinct temporal patterns in CIA rats and underscore the importance of early assessment of skeletal changes in inflammatory arthritis.

## 1 Introduction

Rheumatoid arthritis (RA) is an autoimmune disease with an unclear pathogenesis. The estimated global prevalence ranges from 0.5 to 1.0%, indicating that approximately 500 to 1,000 people per 100,000 are affected by RA worldwide. Current data show that the global burden of RA is increasing and is expected to continue to rise in the future. According to a recent Global Burden of Disease 2021 analysis, the number of patients with rheumatoid arthritis worldwide is expected to increase to approximately 31.7 million by 2050 (1). Since the etiology of RA is unknown, current treatment focuses on controlling symptoms and preventing disease progression, rather than curing the disease (2). The primary causes of disability and loss of labor force in individuals with RA are joint damage and bone destruction, which can lead to severe osteoporosis in the early stages of the disease. In recent years, there has been increasing attention to the prevention and treatment of RA complications, and a 2022 study found that the proportion of secondary osteoporosis (OP) in RA patients was relatively high, and the prevalence of osteoporosis in RA patients was estimated to be 27.6% (95% CI 23.9–31.3%) (3). Advances in conventional synthetic disease-modifying antirheumatic drugs (csDMARDs) and biologic or targeted therapies have substantially improved the control of RA disease activity and slowed structural progression. Nevertheless, skeletal complications, including local erosive damage and systemic bone loss or osteoporosis, remain clinically relevant, and bone loss may still occur in some patients despite treat-to-target management or low disease activity (2, 4, 5).

Collagen-induced arthritis (CIA) is a well-established experimental model that reproduces several key immunological and pathological features of human RA. The model was first described in rats by Trentham et al., who demonstrated that immunization with native type II collagen could induce chronic inflammatory polyarthritis with proliferative synovitis resembling human RA (6). Subsequent studies refined CIA induction protocols and characterized the clinical and pathological phenotypes of CIA in Wistar rats (7). Micro-CT-based investigations further demonstrated progressive alterations in bone volume and trabecular microarchitecture during CIA demonstrated alterations (8). More recently, short-term temporal changes in joint pathology, pain, bone destruction, secondary osteoporosis, and biomechanical properties have also been investigated in CIA rats over a 1–4-week observation period (9). Nevertheless, previous studies have largely focused on model validation, local joint pathology, therapeutic response, or relatively short observation windows, and the extended temporal relationship between trabecular microarchitectural deterioration and impairment of femoral bending strength and vertebral compressive strength remains incompletely characterized. Therefore, the present study evaluated changes in proximal tibial trabecular microarchitecture and bone biomechanics across a 2–10-week observation period following successful CIA establishment, with the aim of characterizing the temporal sequence of bone loss and mechanical impairment and providing an experimental basis for identifying potential windows for early intervention.

## 2 Materials and methods

### 2.1 Laboratory animals and study design

A total of 66 female Wistar rats, aged 7–8 weeks and weighing 170 ± 10 g at study initiation, were provided by the Laboratory Animal Center, Shanghai Institutes for Biological Sciences, Chinese Academy of Sciences. All animals were of specific pathogen-free (SPF) grade and were housed in the animal facility of the Department of Laboratory Animal Science, Shanghai Jiao Tong University School of Medicine. The animals were maintained at a room temperature of 24 ± 2 °C with free access to food and water. All animal experimental procedures were reviewed and approved by the Experimental Animal Ethics Committee of Shanghai University of Traditional Chinese Medicine (approval no. PZSHUTCM2408310009) and were performed in accordance with institutional guidelines for animal welfare and the care and use of laboratory animals. A total of 48 rats underwent collagen-induced arthritis (CIA) induction and were allocated to six experimental groups according to the scheduled observation time points at 2, 3, 4, 6, 8, and 10 weeks after enrollment (*n* = 8 per time point). An additional 18 non-immunized rats served as normal controls. Three normal control rats were euthanized at each corresponding observation time point (2, 3, 4, 6, 8, and 10 weeks; *n* = 3 per time point). Normal control animals received neither type II collagen nor Freund’s adjuvant and were housed under the same environmental conditions as the CIA animals. At each scheduled observation time point, animals were euthanized and tissue specimens were collected immediately. The entire left femur and the fifth lumbar vertebra (L5) were harvested, cleared of surrounding soft tissues, and used for biomechanical testing. The right tibia was collected for a micro-CT assessment of proximal tibial trabecular bone. The same specimen collection strategy was applied to the normal control animals euthanized at the corresponding observation time points. The rats were exposed to CO_2_ in their home cages at a controlled displacement rate of 30–70% container volume per minute. Death was confirmed by ensuring the absence of pedal reflex and respiration, followed by a secondary physical method to ensure permanent cessation of vital signs.

### 2.2 Main reagents

Bovine type II collagen; complete Freund’s adjuvant (CFA); incomplete Freund’s adjuvant (IFA).

### 2.3 Induction of collagen-induced arthritis

The CIA model was established using a type II collagen immunization protocol based on previously described methods, with modifications. For primary immunization, 0.12 mL of bovine type II collagen emulsion containing 240 μg of collagen was injected subcutaneously at the base of the tail. On day 7 after primary immunization, a booster injection of 0.06 mL of collagen emulsion containing a total of 120 μg of collagen was administered subcutaneously into the bilateral dorsal hip regions.

### 2.4 Clinical arthritis assessment, enrollment criteria, and group allocation

Following CIA induction, the severity of arthritis was evaluated macroscopically using a clinical arthritis scoring system based on the extent of paw erythema and swelling, with reference to previously described scoring approaches for rat CIA models (7, 10, 11). Clinical arthritis severity was evaluated by scoring both hind paws separately using a 0–4 scale. A score of 0 indicated no visible signs of arthritis; 1, mild erythema and/or swelling; 2, moderate erythema and swelling involving the ankle joint; 3, marked erythema and swelling extending from the ankle to the metatarsal region; and 4, severe diffuse erythema and swelling involving the entire hind paw, with pronounced joint involvement or functional impairment. The scores of the left and right hind paws were recorded independently, and the mean of the bilateral hind-paw scores was calculated as the clinical arthritis score for each rat.

A mean bilateral hind-paw arthritis score of ≥2 was prespecified as the enrollment threshold for successful CIA establishment. Rats meeting this criterion were considered eligible for inclusion in the CIA experimental groups. Eligible CIA rats were allocated to six experimental groups according to the scheduled specimen-collection time points at 2, 3, 4, 6, 8, and 10 weeks after enrollment (*n* = 8 per time point). At each designated time point, the corresponding animals were euthanized for specimen collection.

In parallel, non-immunized normal control rats were maintained under the same housing conditions and did not receive type II collagen or Freund’s adjuvant. Three normal control rats were euthanized at each corresponding observation time point (2, 3, 4, 6, 8, and 10 weeks; *n* = 3 per time point).

### 2.5 Micro-computed tomography acquisition and trabecular microarchitecture analysis

After euthanasia, the right tibiae were carefully dissected and cleaned of surrounding soft tissues. Five animals from each CIA experimental group and three corresponding age-matched normal control animals at each observation time point were included in the micro-CT analysis. The specimens were mounted vertically along the longitudinal axis in the sample holder and scanned using a GE eXplore Locus SP micro-computed tomography system (GE Healthcare, Milwaukee, WI, USA). Image reconstruction and three-dimensional trabecular morphometric analyses were performed using MicroView version 2.3.2 software with Advance Bone Analysis (GE Healthcare). Images were reconstructed with an isotropic voxel size of 18 μm. For trabecular bone analysis, a region of interest (ROI) was defined within the proximal tibial metaphysis, beginning 1.5 mm distal to the proximal growth plate and extending for 1.0 mm. Following three-dimensional reconstruction, the trabecular bone within the ROI was segmented and analyzed using the Advance Bone Analysis module of MicroView. The following three-dimensional morphometric parameters were quantified: bone mineral density (BMD, mg/cm^3^), trabecular thickness (Tb.Th, mm), trabecular number (Tb. N, mm^−1^), and trabecular separation (Tb. Sp, mm) (Table 1). The remaining animals were allocated to biomechanical testing and other experimental analyses. For the final micro-CT analysis, five CIA specimens were analyzed at each time point, together with three normal-control specimens from the corresponding observation time point.

**Table 1:** Micro-computed tomography-derived three-dimensional morphometric parameters of cancellous bone.

Name symbol unit		
Bone mineral density BMD	BMD	mg/cm^3^
Trabecular thickness	Tb. Th	mm
Trabeculae number	Tb. N	mm^−1^
Trabecular separation	Tb. Sp	mm

### 2.6 Biomechanical testing

Following euthanasia, the entire left femur and the fifth lumbar vertebra (L5) were carefully harvested and cleared of surrounding soft tissues. For the L5 vertebra, the superior and inferior end surfaces were trimmed as necessary to obtain approximately parallel loading surfaces; the vertebral endplates were not intentionally removed. Immediately after preparation, all specimens were wrapped in gauze moistened with 0.9% saline, and biomechanical testing was completed within 2 h of specimen collection. The testing environment was maintained at 37 °C. All biomechanical tests were performed using a Shimadzu EZ-Test universal testing machine (Shimadzu Corporation, Kyoto, Japan) equipped with a 500-N load cell.

For the femoral three-point bending test, the anteroposterior and mediolateral diameters at the midpoint of the femoral shaft were measured, and their mean was recorded as the representative midshaft diameter. Each femur was positioned in a standardized orientation with the femoral head facing upward. The distal side of the lesser trochanter and the supracondylar region were placed on the two supports, providing a support span of 2 cm, and the midpoint of the femoral shaft served as the loading point. A vertical load was applied at a constant displacement rate of 2 mm/min until femoral fracture occurred. The maximum load at fracture was recorded as the primary biomechanical outcome.

For the L5 vertebral compression test, the anteroposterior and mediolateral diameters of the vertebral body were measured and averaged, and vertebral height was also recorded. The prepared vertebral body was positioned between the compression platens with the trimmed superior and inferior surfaces approximately parallel to the loading plates. Because the endplates were retained, the compression test was interpreted as an assessment of whole-vertebral compressive strength rather than isolated trabecular bone strength. Axial compression was applied along the cranio-caudal axis at a constant displacement rate of 2 mm/min. The test endpoint was defined as compression of the vertebral body to one-third of its initial height. The maximum compressive load was recorded as the primary biomechanical outcome.

For the normal reference group, the final numbers of valid specimens included in the biomechanical analyses were *n* = 10 for the femoral three-point bending test and *n* = 6 for the L5 vertebral compression test. The final number of valid CIA specimens varied slightly among time-point groups according to specimen availability and valid test recordings, and the exact sample sizes are reported in the corresponding figure legend.

Although eight CIA animals were initially allocated to each time-point group, the number of specimens available for the final biomechanical analyses was lower in some groups because not all collected specimens yielded valid biomechanical measurements. Only valid measurements were included in the final analysis, and exact group-specific sample sizes are reported in the corresponding figure legend and Supplementary Table S1.

### 2.7 Statistical processing

Statistical analyses were performed using SPSS version 26.0. Approximate normality was assessed using the Shapiro–Wilk test together with visual inspection of Q–Q plots, and homogeneity of variances was evaluated using the Brown–Forsythe test. For BMD and Tb.Th, when parametric assumptions were satisfied, data were analyzed using ordinary two-way ANOVA with disease status and observation time as independent factors, followed by Šídák-adjusted comparisons between the Normal and Model groups within each corresponding time point. These data are presented as mean ± standard deviation (SD). For Tb.N and Tb.Sp, which showed substantial departures from parametric assumptions, the Normal and Model groups were compared within each observation time point using two-sided unpaired Mann–Whitney tests with Holm–Šídák correction for multiple comparisons. These data are presented as median with interquartile range (IQR). For biomechanical outcomes, independent groups were analyzed using ordinary one-way ANOVA when assumptions were satisfied. Because the prespecified comparisons of interest involved each Model time-point group versus the Normal reference group, Dunnett’s multiple-comparisons test was used. Biomechanical data are presented as mean ± SD. All tests were two-sided. Multiplicity-adjusted *p* values are reported for *post hoc* comparisons, and exact p values are provided whenever possible; values below 0.001 are reported as *p* < 0.001. A two-sided *p* < 0.05 was considered statistically significant. Detailed statistical assumptions and analysis methods are provided in Supplementary Table S2.

## 3 Results

### 3.1 Comparison of changes in cancellous bone in the sagittal plane of the upper tibia

**Figure 1:**
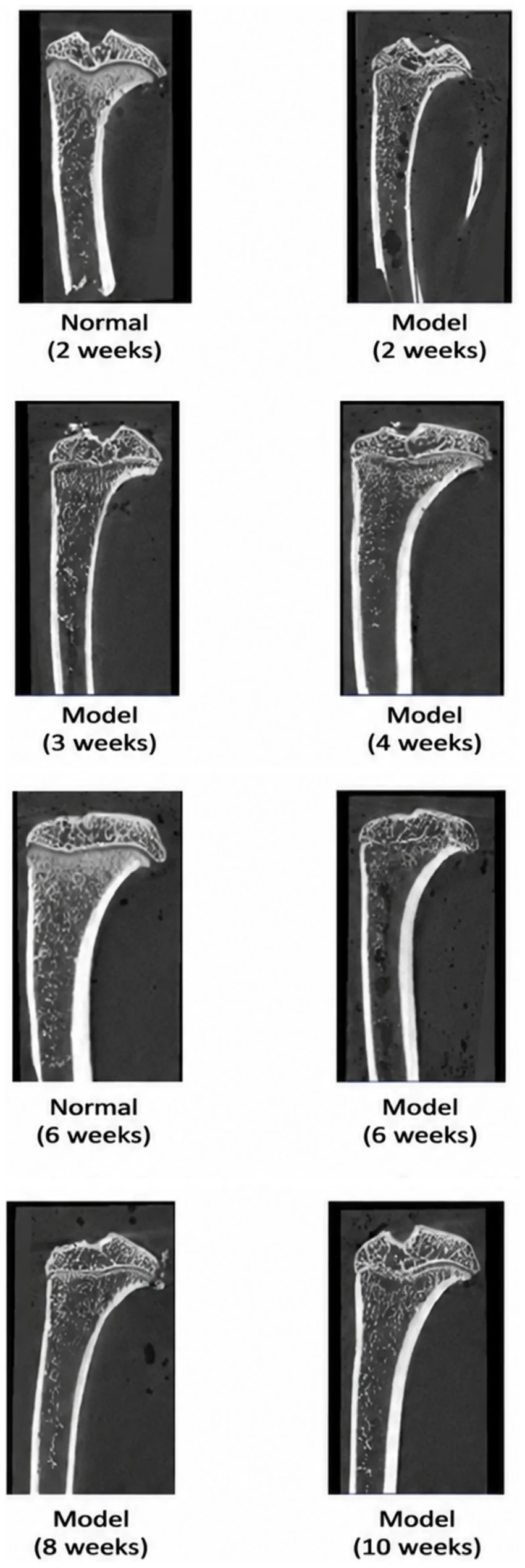
Representative sagittal micro-computed tomography (micro-CT) images of the proximal tibia during collagen-induced arthritis (CIA) progression.

**Figure 2:**
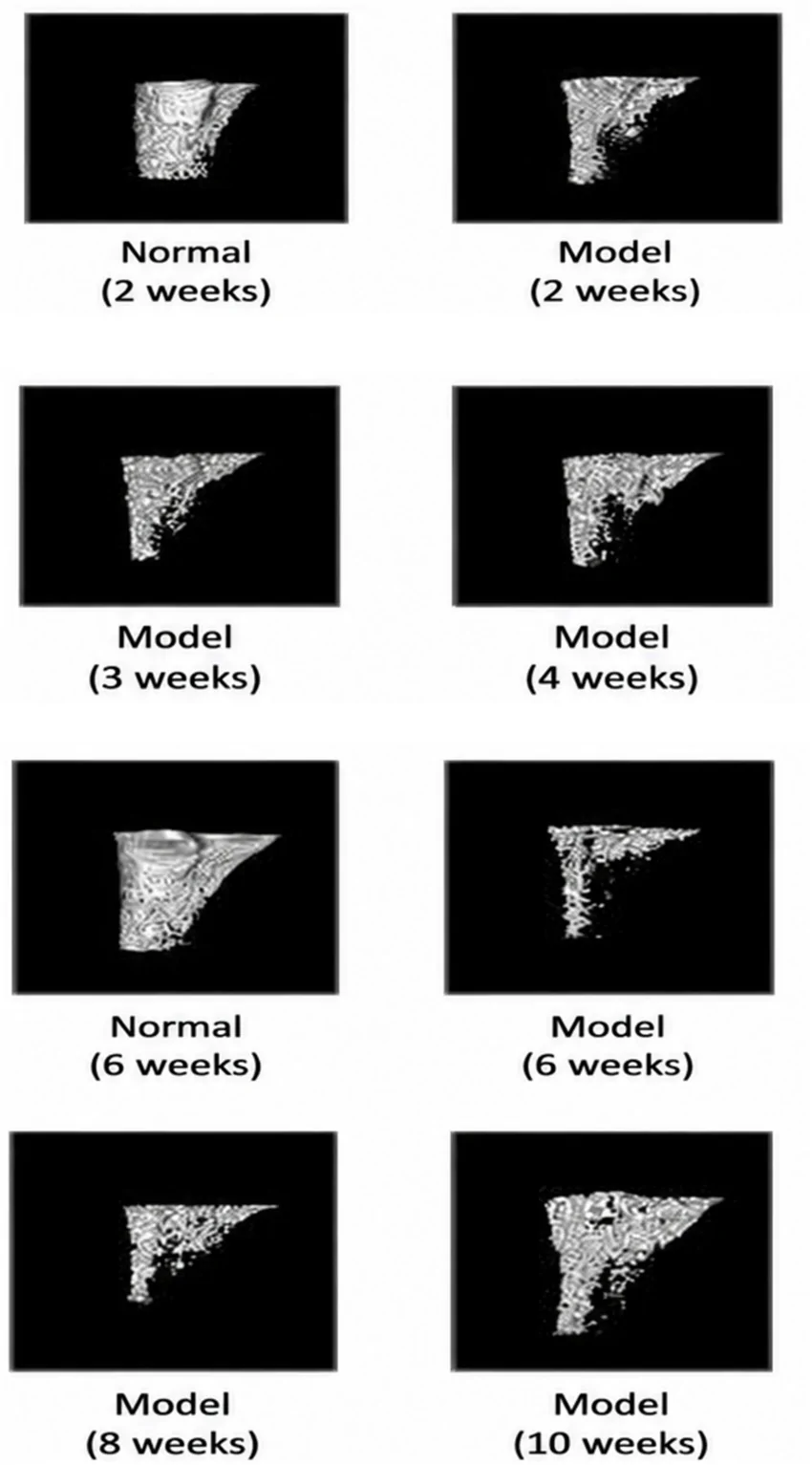
Representative three-dimensional reconstructions of proximal tibial trabecular bone within the predefined region of interest (ROI) during collagen-induced arthritis (CIA) progression.

Micro-CT scans were performed on tibiae from each treatment group to obtain sagittal images of the proximal tibia, as shown in Figures 1, 2. Two weeks after CIA induction, the tibial plateau began to collapse and the epiphyseal line tilted forward. The secondary cancellous trabeculae 1–4 mm below the tibial plateau appeared sparse and disorganized. However, the tibial plateaus of rats in the Normal group largely maintained normal morphology, with relatively preserved trabecular architecture and trabeculae neatly aligned along the direction of vertical stress. Representative images from the later Model groups showed sparse and disorganized secondary cancellous trabeculae.

The proximal tibiae were prepared for micro-CT scanning as described above. Changes in the subchondral bone of the tibial plateau and the trabecular bone in the proximal tibial metaphysis, 1–4 mm distal to the tibial plateau, were compared.

**Figure 3:**
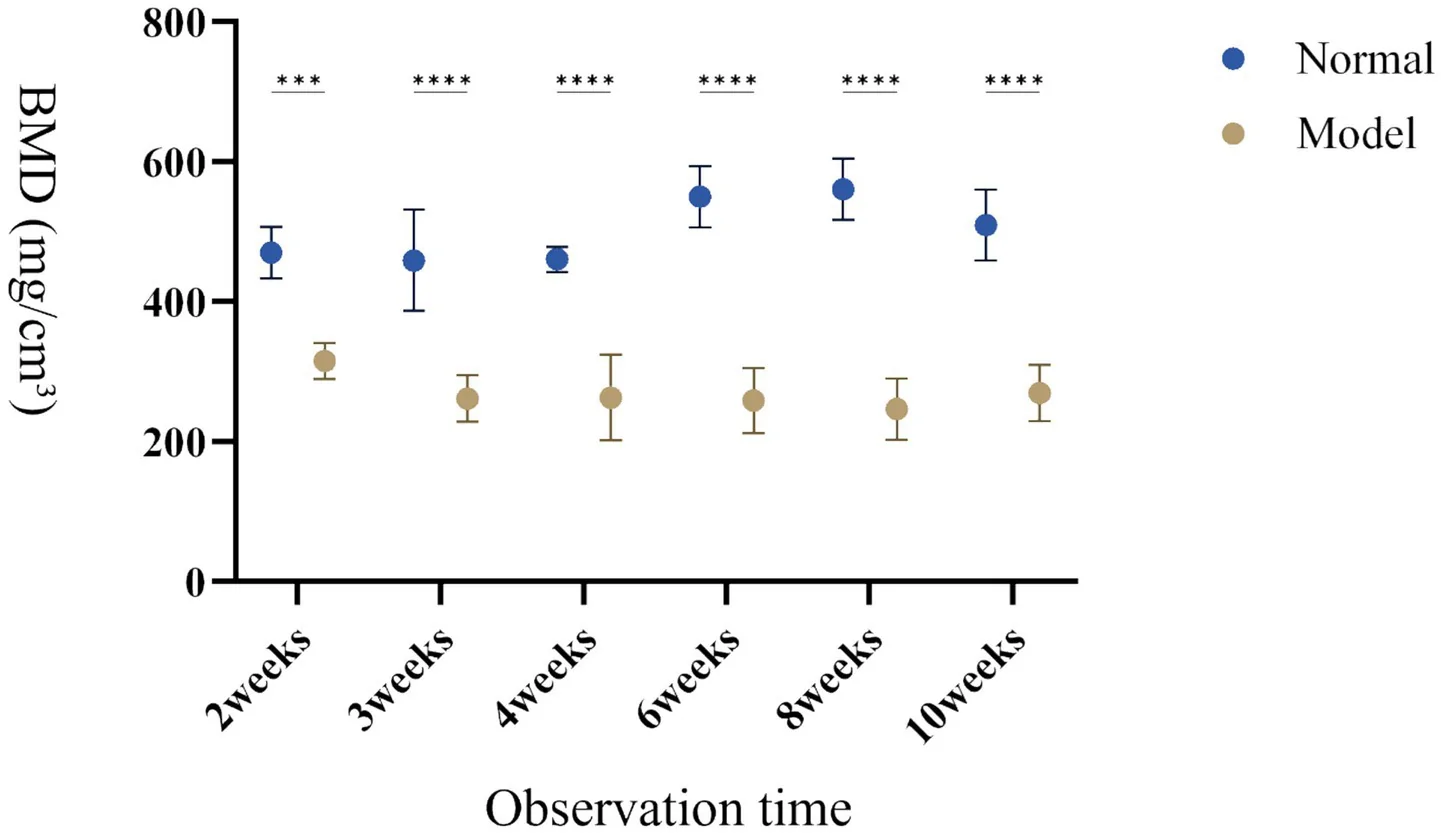
Temporal changes in bone mineral density in the proximal tibial metaphysis of rats during collagen-induced arthritis (CIA) progression. Data are presented as mean ± standard deviation (SD). Statistical comparisons were performed using ordinary two-way analysis of variance (ANOVA) followed by Šídák’s multiple-comparisons test comparing the Normal and Model groups within each corresponding time point. Significance symbols indicate multiplicity-adjusted *p* values. ***p < 0.001; ****p < 0.0001.

Analysis showed that bone mineral density (BMD) differed significantly between the Model group and the corresponding Normal group at 2 weeks after CIA induction and at 3, 4, 6, 8, and 10 weeks (all Šídák-adjusted *p* < 0.001). BMD was significantly reduced at the earliest examined time point after CIA induction, and the reduction remained evident throughout the subsequent observation period (Figure 3).

Panel (A) shows differences in trabecular thickness (Tb.Th). Tb.Th was analyzed using ordinary two-way analysis of variance (ANOVA) followed by Šídák’s multiple-comparisons test, whereas trabecular number (Tb.N) and trabecular separation (Tb.Sp) were analyzed using two-sided Mann–Whitney tests within each time point with Holm–Šídák correction for multiple comparisons.

**Figure 4:**
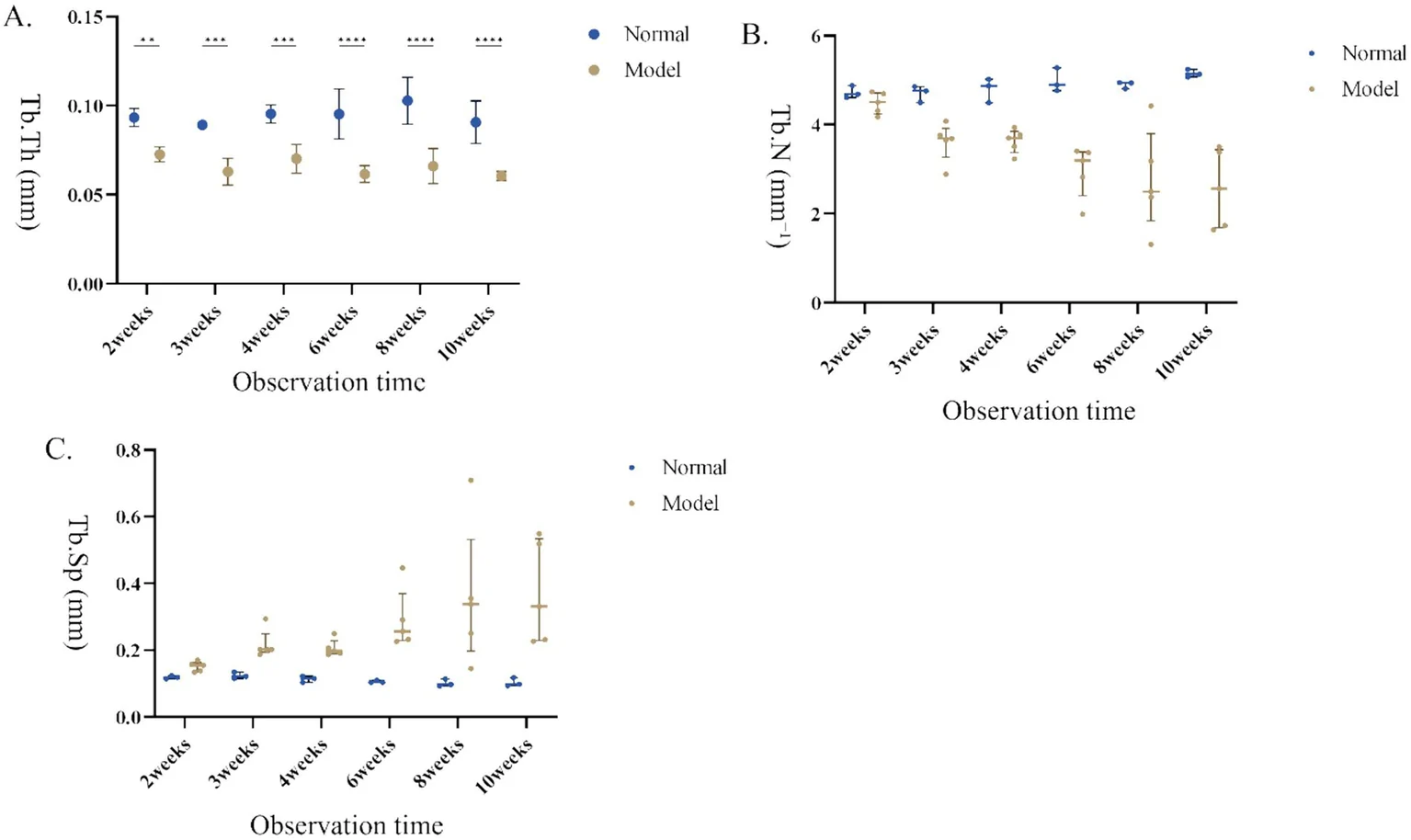
Comparison of trabecular microarchitecture during CIA progression. **(A)** Trabecular thickness (Tb.Th), **(B)** trabecular number (Tb.N), and **(C)** trabecular separation (Tb.Sp). **p < 0.01, ***p < 0.001, and ****p < 0.0001.

As shown in Figure 4A, Tb.Th decreased 2 weeks after CIA induction (adjusted *p* = 0.005) and remained significantly lower than that in the corresponding Normal group at 3, 4, 6, 8, and 10 weeks (all adjusted *p* < 0.001). There was no significant main effect of time and no significant group × time interaction, indicating that no clear progressive temporal change in Tb.Th was detected after the initial reduction.

Figure 4B shows that median Tb.N values in the Model group were numerically lower than those in the corresponding Normal group at several observation time points. However, after Holm–Šídák correction for the six predefined within-time comparisons, none of the differences remained statistically significant. Thus, no statistically significant between-group difference in Tb.N was demonstrated at any individual time point.

Median Tb.Sp values in the Model group were numerically higher than those in the corresponding Normal group at several observation time points. However, after Holm–Šídák correction for the six predefined within-time comparisons, none of the differences remained statistically significant. Thus, no statistically significant between-group difference in Tb.Sp was demonstrated at any individual time point. Detailed Micro-CT results are provided in Supplementary Tables S3 and S4.

### 3.2 Temporal changes in bone biomechanical properties during CIA progression

**Figure 5:**
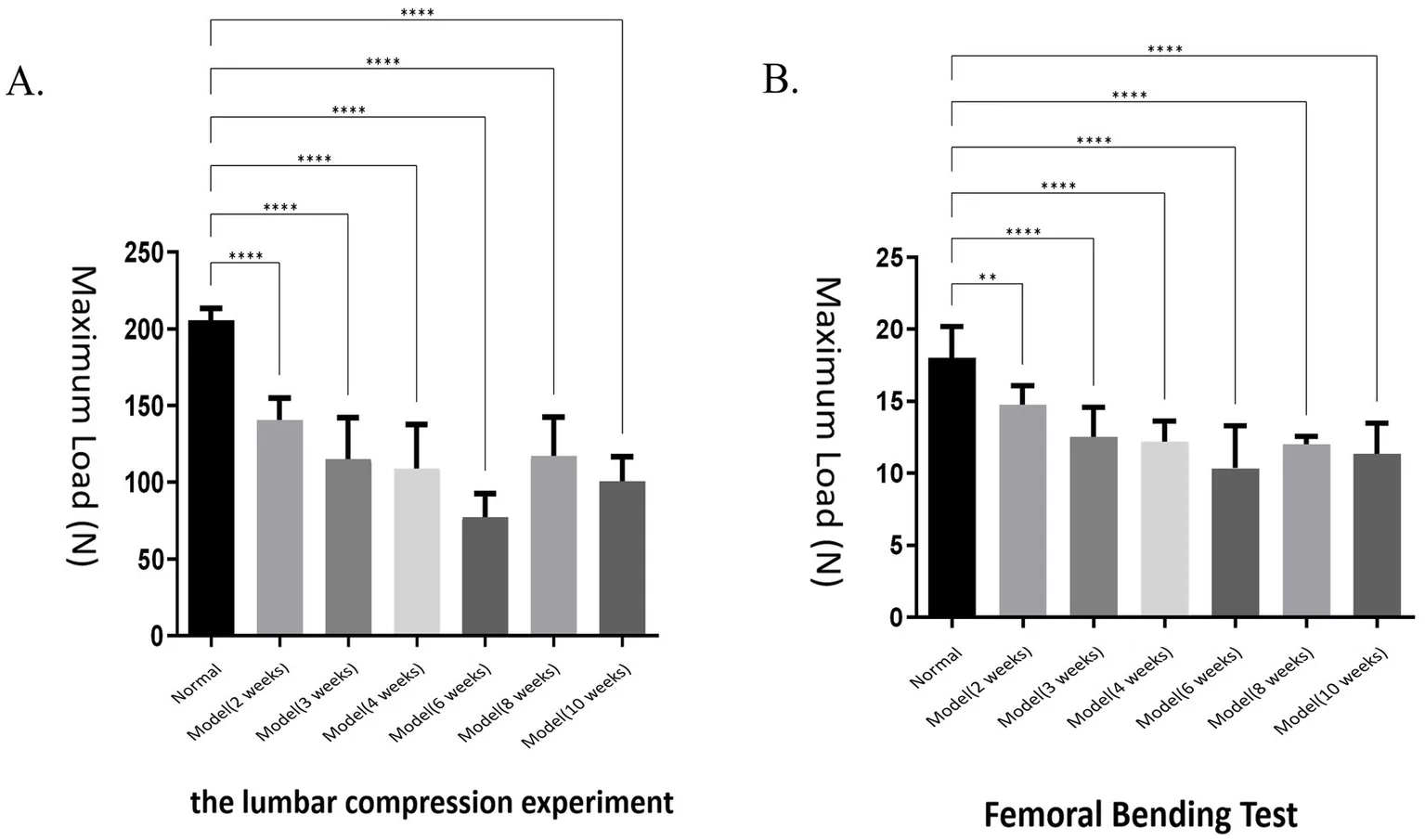
Bone biomechanical properties during collagen-induced arthritis (CIA) progression. **(A)** Maximum compressive load of the fifth lumbar vertebral (L5) body. **(B)** Maximum load in the femoral three-point bending test. Data are presented as mean ± standard deviation (SD). Group differences were analyzed using ordinary one-way analysis of variance (ANOVA) followed by Dunnett’s multiple-comparisons test, with the Normal group specified as the reference control. Reported significance levels correspond to multiplicity-adjusted *p* values. For the L5 vertebral compression test, the Normal group included *n* = 6 animals; Model (2 weeks) and Model (8 weeks) included *n* = 6 each, whereas Model (3 weeks), Model (4 weeks), Model (6 weeks), and Model (10 weeks) included *n* = 7 each. For the femoral three-point bending test, the Normal group included *n* = 10 animals and each model time-point group included *n* = 7 animals. Group-specific sample sizes represent valid biomechanical measurements included in the final analysis. **p < 0.01; ****p < 0.0001.

Bone biomechanical properties differed significantly among groups for both the L5 vertebral compression test and the femoral three-point bending test. For L5 vertebral compressive strength, ordinary one-way ANOVA showed a significant overall group effect (*F* = 24.435, *p* < 0.001). Compared with the Normal group, vertebral compressive strength was significantly reduced in the Model (2 weeks) group (Dunnett-adjusted *p* < 0.001) and remained significantly lower in the Model (3 weeks), Model (4 weeks), Model (6 weeks), Model (8 weeks), and Model (10 weeks) groups (all adjusted *p* < 0.001) (Figure 5A).

Femoral bending strength also differed significantly among groups (ordinary one-way ANOVA, *F* = 15.218, *p* < 0.001). Compared with the Normal group, femoral bending strength was significantly reduced in the Model (2 weeks) group (Dunnett-adjusted *p* = 0.009) and remained significantly lower in the Model (3 weeks), Model (4 weeks), Model (6 weeks), Model (8 weeks), and Model (10 weeks) groups (all adjusted *p* < 0.001) (Figure 5B).

Collectively, these findings indicate that both vertebral compressive strength and femoral bending strength were already impaired at the earliest examined model time point and remained reduced through the final 10-week observation point. However, because the biomechanical analysis used a pooled Normal reference group, the temporal pattern should be interpreted as sustained mechanical impairment relative to the reference condition rather than as evidence of irreversible loss.

## 4 Discussion

The present study characterized the temporal progression of trabecular microarchitectural deterioration and mechanical impairment in a rat CIA model over a 2–10-week observation period. The principal findings were that significant reductions in BMD and Tb.Th were detectable at an early stage, whereas neither Tb.N nor Tb.Sp differed significantly between the Model and corresponding Normal groups at any individual time point after multiplicity correction. In parallel, biomechanical testing demonstrated reductions in both femoral bending strength and vertebral compressive strength. Together, these findings suggest that structural deterioration of trabecular bone and impairment of bone mechanical competence may follow partly distinct temporal patterns during CIA progression. Significant changes in BMD and Tb.Th, despite non-significant time-specific comparisons for Tb.N and Tb.Sp, underscore that mineral-density and trabecular microarchitectural indices provide related but non-identical information. These observations may have implications for identifying earlier windows for the assessment and prevention of skeletal complications associated with inflammatory arthritis.

BMD and trabecular microarchitectural indices provide complementary but distinct information on skeletal status. BMD is a Micro-CT-derived mineral-density parameter reflecting mineralization within the selected region of interest, whereas Tb.Th, Tb.N, and Tb.Sp characterize different aspects of trabecular architecture. Under simplified stereological assumptions, the product of trabecular thickness and trabecular number is more closely related to trabecular bone volume fraction (BV/TV) than to BMD. Accordingly, changes in BMD should not be interpreted as mathematically equivalent to alterations in trabecular thickness or number. In the present study, the early and sustained reduction in BMD, together with changes in trabecular microarchitectural indices, supports the interpretation that mineral-density loss and structural alteration represent related but non-identical components of CIA-associated skeletal deterioration (4).

Despite substantial advances in the treatment of RA with conventional disease-modifying antirheumatic drugs and biologic agents, skeletal complications remain clinically relevant. Some patients continue to experience local joint damage or systemic bone loss despite improved control of disease activity. This clinical discrepancy highlights the need to better understand the temporal relationship between inflammatory disease progression, deterioration of bone microarchitecture, and loss of mechanical competence.

Clinical evidence consistently demonstrates an increased burden of osteoporosis and fragility fractures in patients with RA. Kim et al. (12) reported an approximately twofold higher incidence of fragility fractures in patients with RA than in the general population. In China, pooled analyses have estimated the prevalence of osteoporosis among patients with RA at approximately 30–40%, with substantial regional variation (13, 14). A multicenter meta-analysis involving 12,264 Chinese patients with RA reported a pooled osteoporosis frequency of 37.67%, whereas a hospital-based cohort of 549 patients identified osteoporosis in 33.3% and vertebral fractures in 25.7% (13, 15). The prevalence of OP among patients with RA is generally high but varies across regions (16). This variation may be related to geographic factors and the baseline characteristics of the patients with RA included in the studies. Previous studies have shown that osteoporosis secondary to RA is significantly associated with disease activity, suggesting that patients with early RA should undergo osteoporosis assessment and receive active disease control (5, 17, 18). Several studies have shown that older age is an independent risk factor for secondary OP (16, 19). The risk depends on several factors, including age, sex, disease activity, and treatment (5, 19). Studies have shown that older patients with RA generally have lower bone mineral density and a higher prevalence of OP (17, 20, 21). Specifically, older age, female sex, and hormonal changes (e.g., postmenopause) increase the risk of osteoporosis in patients with RA (13, 15, 19).

The temporal pattern observed in the present study suggests that CIA-associated skeletal changes cannot be fully characterized by BMD alone. Early reductions in BMD and Tb.Th were statistically significant, whereas comparisons of Tb.N and Tb.Sp between the Model and corresponding Normal groups were not significant at any individual time point after multiplicity correction. Accordingly, Tb.N and Tb.Sp should not be interpreted as demonstrating progressive change in the present dataset. Nevertheless, because these indices characterize aspects of trabecular architecture distinct from mineral density, they remain relevant outcomes for future longitudinal studies with adequate power to detect time-dependent effects. Tb.Sp reflects the average distance between adjacent trabeculae and is commonly used as an indicator of trabecular structural organization. Higher Tb.Sp values generally indicate greater separation between trabeculae and a sparser trabecular architecture. In the present study, the numerical differences in Tb.Sp did not translate into statistically significant between-group differences after correction and therefore cannot support a conclusion of time-specific structural change.

In recent years, it has been suggested that ectopic germinal centers (EGCs) may be an important cause of local bone destruction and osteoporosis in RA (22–24). EGCs occur in the target organs of numerous autoimmune diseases and, in RA, are mainly found in the synovium of affected joints (22, 23, 25). EGCs locally produce specific autoantibodies that maintain the immune response and are closely associated with disease severity and treatment sensitivity (23, 26–28). EGCs in the RA synovium can induce B-cell affinity maturation and differentiation into plasma cells. The resulting response to anticitrullinated antigens can induce local tissue-specific pathogenic autoantibodies, directly leading to local joint inflammation (23, 26, 28, 29). This process accelerates the destruction of joint cartilage and subchondral bone (24, 25, 30).

In addition, numerous chemokines and inflammatory factors, including interleukin (IL)-17, IL-21, lymphotoxin alpha/beta (LTα/*β*), inducible T-cell co-stimulator (ICOS)–ICOS ligand (ICOSL), and B-cell activating factor (BAFF), play roles in the initiation, formation, and maintenance of EGCs (22, 31–33). However, the mechanisms that inhibit EGC formation have not been investigated further; therefore, future research should focus on this topic (22, 34).

Several limitations should be considered when interpreting the present findings. First, inflammatory activity was not longitudinally quantified at each observation time point using serial clinical arthritis scores, paw-swelling measurements, or histological synovitis scores. Therefore, the temporal relationship between changes in inflammatory activity and skeletal deterioration could not be directly established. Second, the biomechanical analyses used a pooled Normal reference group because the final numbers of valid normal specimens differed between the femoral three-point bending and L5 vertebral compression tests. Accordingly, the biomechanical findings should be interpreted as sustained impairment relative to the normal reference condition rather than as evidence of irreversible mechanical loss or as fully time-matched longitudinal trajectories. Third, the relatively small numbers of time-matched normal animals in the Micro-CT analysis reduced statistical power, particularly for non-parametric comparisons of Tb.N and Tb.Sp. These limitations should be considered when interpreting the temporal patterns observed in the present study.

In conclusion, this study characterized temporal changes in bone loss, trabecular microarchitecture, and mechanical competence in a rat CIA model over a 2–10-week observation period. Micro-CT analysis demonstrated early and sustained reductions in BMD and Tb.Th; no statistically significant between-group differences in Tb.N or Tb.Sp were identified at individual time points after multiplicity correction. Together, these findings indicate that skeletal deterioration is detectable early during CIA progression and may involve partly distinct changes in bone mineral density, trabecular architecture, and mechanical competence. The results support the importance of early skeletal assessment in inflammatory arthritis and provide an experimental basis for future studies aimed at preserving bone structure and strength.

## Data Availability

The original contributions presented in the study are included in the article and its Supplementary material. Further inquiries can be directed to the corresponding author.
