# Supplementary material for: Temporal progression of bone loss, trabecular microarchitectural deterioration, and mechanical impairment in a rat model of collagen-induced arthritis
Source: Front Med (Lausanne). 2026 Aug 17;13:1885782. doi: 10.3389/fmed.2026.1885782 (PMC13527451; doi:10.3389/fmed.2026.1885782)
Supplement: Supplementary file 1 [file Table_1.docx]

**Supplementary Statistical Tables**

**Supplementary Table S1. Descriptive statistics, 95% confidence intervals, and Dunnett-adjusted comparisons for biomechanical outcomes**

**A. Femoral three-point bending strength**

| **Group** | **n** | **Mean ± SD (N)** | **95% CI of mean (N)** | **Dunnett-adjusted P vs Normal** |
| --- | --- | --- | --- | --- |
| Normal | 10 | 18.02 ± 2.16 | 16.47 to 19.57 | — |
| Model (2 weeks) | 7 | 14.77 ± 1.31 | 13.56 to 15.98 | 0.009 |
| Model (3 weeks) | 7 | 12.53 ± 2.07 | 10.62 to 14.44 | 5.076E-06 |
| Model (4 weeks) | 7 | 12.20 ± 1.42 | 10.89 to 13.51 | 1.398E-06 |
| Model (6 weeks) | 7 | 10.34 ± 2.97 | 7.59 to 13.09 | 1.649E-08 |
| Model (8 weeks) | 7 | 12.01 ± 0.54 | 11.51 to 12.51 | 2.079E-06 |
| Model (10 weeks) | 7 | 11.35 ± 2.15 | 9.36 to 13.34 | 5.391E-08 |

**B. L5 vertebral compressive strength**

| **Group** | **n** | **Mean ± SD (N)** | **95% CI of mean (N)** | **Dunnett-adjusted P vs Normal** |
| --- | --- | --- | --- | --- |
| Normal | 6 | 205.51 ± 7.86 | 197.26 to 213.76 | — |
| Model (2 weeks) | 6 | 140.74 ± 14.43 | 125.60 to 155.88 | 1.674E-05 |
| Model (3 weeks) | 7 | 115.29 ± 26.89 | 90.42 to 140.16 | 1.718E-09 |
| Model (4 weeks) | 7 | 109.02 ± 28.49 | 82.67 to 135.37 | 2.326E-10 |
| Model (6 weeks) | 7 | 77.42 ± 15.42 | 63.16 to 91.68 | 2.853E-14 |
| Model (8 weeks) | 6 | 117.37 ± 25.08 | 91.05 to 143.69 | 1.873E-06 |
| Model (10 weeks) | 7 | 100.68 ± 16.01 | 85.87 to 115.49 | 5.582E-11 |

***Note.*** Data are presented as mean ± SD. The 95% CI is a two-sided confidence interval for the group mean. Overall group differences were assessed using ordinary one-way ANOVA (femoral bending: F(6,45)=15.218, P=1.995E-09; L5 compression: F(6,39)=24.435, P=8.661E-12), followed by Dunnett's multiple-comparisons test comparing each CIA time-point group with the pooled Normal reference group. Reported pairwise P values are multiplicity-adjusted and two-sided. Sample sizes represent valid specimens included in the final analysis and may differ from the initially allocated n=8 per CIA time-point group.

**Supplementary Table S2. Assumption checks, data presentation, and statistical analysis plan for biomechanical and Micro-CT outcomes**

| **Outcome** | **Data presentation** | **Normality assessment** | **Variance homogeneity** | **Primary analysis** | **Multiplicity control** |
| --- | --- | --- | --- | --- | --- |
| Femoral bending strength | Mean ± SD | Shapiro–Wilk: all groups P>0.05; Q–Q plots acceptable | Brown–Forsythe P=0.439 | Ordinary one-way ANOVA | Dunnett vs pooled Normal |
| L5 vertebral compression | Mean ± SD | Shapiro–Wilk: all groups P>0.05; Q–Q plots acceptable | Brown–Forsythe P=0.325 | Ordinary one-way ANOVA | Dunnett vs pooled Normal |
| BMD | Mean ± SD | Model residuals: Shapiro–Wilk P=0.617; Q–Q plots acceptable | Brown–Forsythe P=0.946 | Ordinary two-way ANOVA | Šídák within-time Normal vs Model |
| Tb.Th | Mean ± SD | Model residuals: Shapiro–Wilk P=0.585; Q–Q plots acceptable | Brown–Forsythe P=0.728 | Ordinary two-way ANOVA | Šídák within-time Normal vs Model |
| Tb.N | Median (IQR) | Model residuals: Shapiro–Wilk P=0.003 | Brown–Forsythe P=0.074 | Two-sided unpaired Mann–Whitney tests within time points | Holm–Šídák across six time-point comparisons |
| Tb.Sp | Median (IQR) | Model residuals: Shapiro–Wilk P<0.001 | Brown–Forsythe P=0.030 | Two-sided unpaired Mann–Whitney tests within time points | Holm–Šídák across six time-point comparisons |

***Note.*** Approximate normality was evaluated using the Shapiro–Wilk test together with visual inspection of Q–Q plots. For the two-way ANOVA models, normality was assessed on model residuals. Homogeneity of variances was evaluated using the Brown–Forsythe test. Because Tb.N and Tb.Sp did not adequately satisfy parametric assumptions, these outcomes were summarized using median (IQR) and analyzed using nonparametric within-time comparisons. All tests were two-sided.

**Supplementary Table S3. Parametric Micro-CT outcomes: descriptive statistics, 95% confidence intervals, two-way ANOVA effects, and Šídák-adjusted within-time comparisons**

**A. Bone mineral density (BMD)**

| **Time point** | **Normal n** | **Normal mean ± SD (mg/cm³)** | **Normal 95% CI (mg/cm³)** | **Model n** | **Model mean ± SD (mg/cm³)** | **Model 95% CI (mg/cm³)** | **Šídák-adjusted P** |
| --- | --- | --- | --- | --- | --- | --- | --- |
| 2 weeks | 3 | 469.38 ± 37.09 | 377.25 to 561.52 | 5 | 315.18 ± 26.04 | 282.85 to 347.51 | 0.000208 |
| 3 weeks | 3 | 458.48 ± 72.22 | 279.08 to 637.88 | 5 | 261.06 ± 33.02 | 220.06 to 302.06 | 3.572E-06 |
| 4 weeks | 3 | 460.10 ± 18.27 | 414.70 to 505.49 | 5 | 262.48 ± 61.77 | 185.78 to 339.17 | 3.506E-06 |
| 6 weeks | 3 | 549.42 ± 43.53 | 441.27 to 657.56 | 5 | 258.26 ± 46.60 | 200.40 to 316.13 | 7.144E-10 |
| 8 weeks | 3 | 560.58 ± 44.05 | 451.15 to 670.01 | 5 | 246.44 ± 43.93 | 191.89 to 300.99 | 1.018E-10 |
| 10 weeks | 3 | 509.01 ± 50.86 | 382.66 to 635.35 | 5 | 269.21 ± 40.10 | 219.42 to 319.00 | 6.900E-08 |

**B. Trabecular thickness (Tb.Th)**

| **Time point** | **Normal n** | **Normal mean ± SD (mm)** | **Normal 95% CI (mm)** | **Model n** | **Model mean ± SD (mm)** | **Model 95% CI (mm)** | **Šídák-adjusted P** |
| --- | --- | --- | --- | --- | --- | --- | --- |
| 2 weeks | 3 | 0.09330 ± 0.00497 | 0.08095 to 0.10565 | 5 | 0.07261 ± 0.00416 | 0.06744 to 0.07778 | 0.005 |
| 3 weeks | 3 | 0.08916 ± 0.00190 | 0.08444 to 0.09388 | 5 | 0.06295 ± 0.00750 | 0.05364 to 0.07226 | 0.000298 |
| 4 weeks | 3 | 0.09539 ± 0.00519 | 0.08251 to 0.10828 | 5 | 0.07014 ± 0.00802 | 0.06019 to 0.08010 | 0.000494 |
| 6 weeks | 3 | 0.09520 ± 0.01401 | 0.06041 to 0.12999 | 5 | 0.06151 ± 0.00463 | 0.05576 to 0.06727 | 5.324E-06 |
| 8 weeks | 3 | 0.10283 ± 0.01307 | 0.07035 to 0.13531 | 5 | 0.06605 ± 0.00982 | 0.05385 to 0.07824 | 1.003E-06 |
| 10 weeks | 3 | 0.09069 ± 0.01202 | 0.06083 to 0.12055 | 5 | 0.06060 ± 0.00263 | 0.05732 to 0.06387 | 3.710E-05 |

**C. Overall two-way ANOVA effects**

| **Outcome** | **Effect** | **df1** | **df2** | **F** | **P** |
| --- | --- | --- | --- | --- | --- |
| BMD | Disease status (Group) | 1 | 36 | 304.335 | 3.827E-19 |
| BMD | Observation time | 5 | 36 | 1.051 | 0.403 |
| BMD | Group × Time interaction | 5 | 36 | 3.519 | 0.011 |
| Tb.Th | Disease status (Group) | 1 | 36 | 153.573 | 1.504E-14 |
| Tb.Th | Observation time | 5 | 36 | 2.017 | 0.100 |
| Tb.Th | Group × Time interaction | 5 | 36 | 1.077 | 0.389 |

***Note.*** Data are presented as mean ± SD. The 95% CI is a two-sided confidence interval for the group mean and is provided as descriptive information; it is not the confidence interval for the between-group difference. Ordinary two-way ANOVA included disease status (Normal vs Model), observation time, and their interaction. Within each time point, Normal and Model groups were compared using Šídák-adjusted post hoc tests. Reported pairwise P values are multiplicity-adjusted and two-sided. BMD, bone mineral density; Tb.Th, trabecular thickness.

**Supplementary Table S4. Nonparametric Micro-CT outcomes: medians, interquartile ranges, Mann–Whitney statistics, and Holm–Šídák-adjusted comparisons**

**A. Trabecular number (Tb.N)**

| **Time point** | **Normal n** | **Normal median (IQR) (mm⁻¹)** | **Model n** | **Model median (IQR) (mm⁻¹)** | **Mann–Whitney U** | **Raw P** | **Holm–Šídák adjusted P** |
| --- | --- | --- | --- | --- | --- | --- | --- |
| 2 weeks | 3 | 4.680 (4.644–4.779) | 5 | 4.506 (4.312–4.693) | 11 | 0.393 | 0.393 |
| 3 weeks | 3 | 4.757 (4.628–4.805) | 5 | 3.687 (3.650–3.764) | 15 | 0.036 | 0.196 |
| 4 weeks | 3 | 4.872 (4.682–4.945) | 5 | 3.695 (3.508–3.767) | 15 | 0.036 | 0.196 |
| 6 weeks | 3 | 4.891 (4.826–5.084) | 5 | 3.191 (2.825–3.371) | 15 | 0.036 | 0.196 |
| 8 weeks | 3 | 4.936 (4.872–4.938) | 5 | 2.496 (2.373–3.177) | 15 | 0.036 | 0.196 |
| 10 weeks | 3 | 5.133 (5.100–5.189) | 5 | 2.562 (1.734–3.377) | 15 | 0.036 | 0.196 |

**B. Trabecular separation (Tb.Sp)**

| **Time point** | **Normal n** | **Normal median (IQR) (mm)** | **Model n** | **Model median (IQR) (mm)** | **Mann–Whitney U** | **Raw P** | **Holm–Šídák adjusted P** |
| --- | --- | --- | --- | --- | --- | --- | --- |
| 2 weeks | 3 | 0.118 (0.116–0.121) | 5 | 0.155 (0.138–0.157) | 0 | 0.036 | 0.196 |
| 3 weeks | 3 | 0.122 (0.118–0.128) | 5 | 0.202 (0.202–0.203) | 0 | 0.036 | 0.196 |
| 4 weeks | 3 | 0.115 (0.109–0.119) | 5 | 0.198 (0.192–0.207) | 0 | 0.036 | 0.196 |
| 6 weeks | 3 | 0.105 (0.104–0.107) | 5 | 0.256 (0.232–0.291) | 0 | 0.036 | 0.196 |
| 8 weeks | 3 | 0.097 (0.095–0.106) | 5 | 0.338 (0.250–0.355) | 0 | 0.036 | 0.196 |
| 10 weeks | 3 | 0.099 (0.096–0.108) | 5 | 0.331 (0.231–0.518) | 0 | 0.036 | 0.196 |

***Note.*** Data are presented as median (IQR), where IQR denotes the 25th–75th percentile. Normal and Model groups were compared within each observation time point using two-sided unpaired Mann–Whitney tests. Raw P values were adjusted across the six predefined time-point comparisons for each outcome using the Holm–Šídák procedure. No global two-way ANOVA was reported for Tb.N or Tb.Sp because parametric assumptions were not adequately satisfied. Tb.N, trabecular number; Tb.Sp, trabecular separation.
